# High-Performance Core/Shell of ZnO/TiO_2_ Nanowire with AgCl-Doped CdSe Quantum Dots Arrays as Electron Transport Layer for Perovskite Solar Cells

**DOI:** 10.3390/molecules25173969

**Published:** 2020-08-31

**Authors:** Jin Mo Kim, Bong Soo Lee, Sung Won Hwang

**Affiliations:** 1 Micro LED Research Center, Korea Photonics Technology Institute, Gwangju 61007, Korea; jmkim@kopti.re.kr; 2 Department of Energy Systems Engineering, Chung Ang University, Seoul 06974, Korea; 3 Department of System Semiconductor Engineering, Sangmyung University, 31 Sangmyung dae-gil, Cheonan 31066, Korea

**Keywords:** ZnO/TiO_2_ nanowire, AgCl-doped CdSe quantum dots, electron transport layer, perovskite solar cells, carrier transport

## Abstract

Most previous studies of perovskite core/shell structures have been based on ZnO/TiO_2_ nanowires (NWs), which are not suitable for high photoelectric conversion efficiency. Here, core/shell ZnO/TiO_2_ NWs with AgCl-doped CdSe quantum dots were fabricated as an electron transport layer (ETL) for perovskite solar cells, based on ZnO/TiO_2_ arrays. We designed CdSe with AgCl dopants that were synthesized by a colloidal process. An improvement of the recombination barrier (R_ct1_), due to shell supplementation with AgCl-doped CdSe quantum dots, improved the open circuit voltage, the fill factor, and the adsorption capacity of CH_3_NH_3_PbI_3_ perovskite with NWs. The enhanced cell steady state was attributable to TiO_2_ with AgCl-doped CdSe QD supplementation. A maximum power conversion efficiency of 15.12% was attained in an atmospheric environment. The mechanism of the recombination and electron transport in the perovskite solar cells becoming the basis of ZnO/TiO_2_ core/shell arrays was investigated to represent the merit of ZnO/TiO_2_ core/shell arrays as an electron transport layer in effective devices. These results showed an uncomplicated approach for restraining non-radiative recombination loss in hetero-structure core/shell arrays to significantly improve perovskite solar cell performance and increase the effectiveness of photovoltaics.

## 1. Introduction

Perovskite photovoltaics based on the combination of organo–lead halide photon energies have gained intensive interest owing to their excellent photoelectric efficiencies and potential for use in flexible devices. For example, the perovskite CH_3_NH_3_PbI_3_ has the merits of high mobility and carrier diffusion and a long lifetime [1,2,3,4,5,6,7,8]. To improve the device stability and performance of perovskite photovoltaics, studies have proposed several strategies such as a CdSe quantum dot (QD)/PCBM hybrid for improved carrier diffusion [9,10], changing the iodide ion concentration [11], introducing a SnO_2_ protective structure between perovskite layers and ZnO nanowires (NWs) with TiO_2_ modification for carrier recombination [12,13,14]. However, previous research has shown that a decrease in the carrier recombination of TiO_2_ occurs owing to the mismatched electron recombination of perovskite photovoltaics [15,16]. With its wide direct band gap (*E*_g_ = 3.37 eV at room temperature) [17,18], ZnO exhibits a different structure in photovoltaics [19,20,21] and is considered a promising alternative to the present TiO_2_ arrays with AgCl-doped CdSe QDs, characterized by rapid carrier recombination [22,23]. Furthermore, ZnO/TiO_2_ with AgCl-doped CdSe QDs as an electron transport layer (ETL) has the advantage of improving the photon absorption and thus presenting a significant path for carrier recombination. Although the photoelectric conversion efficiency of ZnO-based devices is significantly lower than that of TiO_2_-based cells, the carrier transport leads to a low charge recombination of ZnO and the stability of perovskite/zinc heterostructures [24,25].

In this work, TiO_2_ arrays with AgCl-doped CdSe QDs were designed as the ETL for perovskite solar cells. We investigate perovskite photovoltaics based on zinc heterostructure core/shell arrays with AgCl-doped CdSe QDs in an atmospheric environment. The hybrid heterostructure of TiO_2_, with AgCl-doped CdSe QDs was analyzed via scanning electron microscopy (SEM) and transmission electron microscopy (TEM), and the morphology of the ZnO/TiO_2_ core/shell arrays was compared by field emission SEM (FE-SEM) and high-resolution TEM (HR-TEM). In addition, the effects of modification by TiO_2_ with AgCl-doped CdSe QDs on the structural and optical properties of CH_3_NH_3_PbI_3_ layers on ZnO NWs were studied. The photoelectric conversion efficiency, stability, and power conversion efficiency of perovskite solar cells fabricated with ZnO NWs were determined via SEM, TEM, incident photon conversion efficiency (IPCE), photocurrent density–voltage (J–V) analysis, and electrochemical impedance spectroscopy. The mechanism of electron–hole recombination centers in perovskite solar cells based on the ZnO/TiO_2_ core/shell arrays with AgCl-doped CdSe QDs was also discussed.

## 2. Results and Discussion

### 2.1. SEM and HRTEM Studies

**Figure 1:**
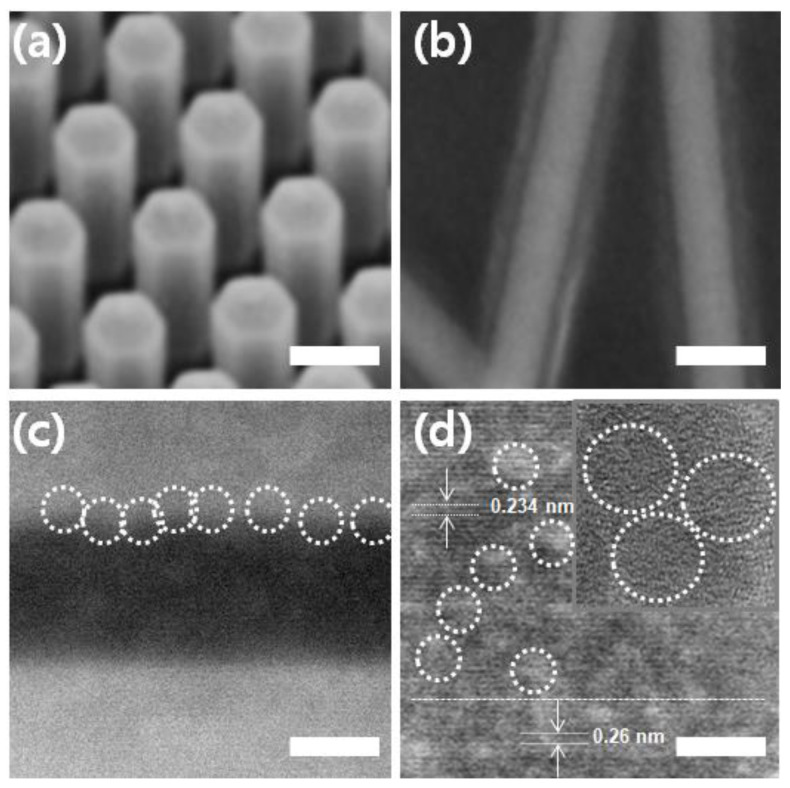
(**a**) Top view SEM images of the nanowires (NWs) of ZnO/TiO_2_, the scale bar indicates 1 μm; (**b**) HR-TEM image of ZnO/TiO_2_ core/shell NWs, the scale bar indicates 500 nm; (**c**) high magnification cross-sectional TEM images of the NWs with AgCl-doped CdSe quantum dot (QD) interlayer, the scale bar indicates 50 nm; (**d**) and a high-resolution TEM image (white circle) of QDs, the scale bar indicates 10 nm.

**Figure 2:**
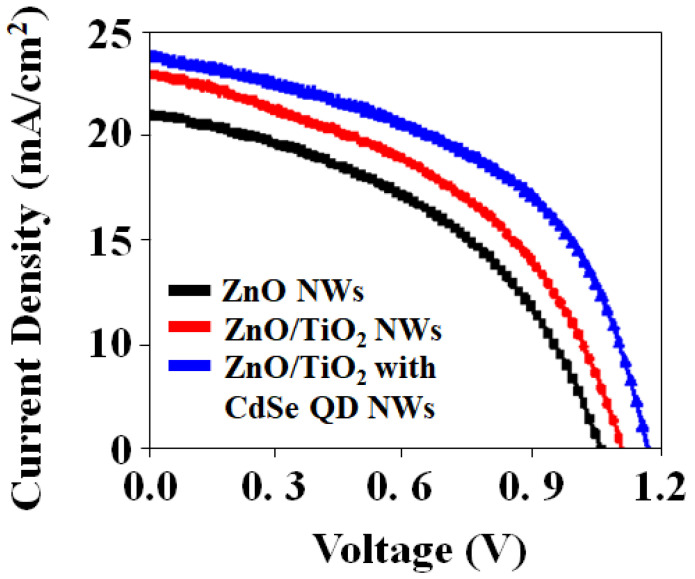
Photocurrent density–voltage (J–V) characteristics of perovskite solar cells (PSCs) based on ZnO/TiO_2_ core/shell arrays with AgCl-doped CdSe quantum dots under 100 mW/cm^2^ of AM 1.5 illumination.

Figure 1 presents a cross-sectional SEM image of a perovskite solar cell based on ZnO/TiO_2_ NWs. It is clear that the heterogeneous perovskite modification by ZnO/TiO_2_ with AgCl-doped CdSe QDs entirely coated the structures between the NWs. Figure 1 shows that the ZnO/TiO_2_ NWs, which are significant in that the carriers transported after photon on the topside overspreading perovskite CH_3_NH_3_PbI_3_ heterogeneous with the AgCl-doped CdSe QDs in the surface of NWs, had a great point of contact area with the ZnO/TiO_2_ NWs and were promptly transported through the ZnO NWs to the electrode region, therefore decreasing the recombination centers and enhancing the photoelectric conversion efficiency. The SEM and HR-TEM images in Figure 1a,b, respectively, indicate that the cell design resulted in TiO_2_ modification with appropriate coverage of the ZnO NWs. The SEM image of the ZnO/TiO_2_ NWs in Figure 2 indicates that the heterostructure was uniformly coated by 1 µm ZnO/TiO_2_ NWs. Figure 1b presents a cross-sectional HR-TEM image of the ZnO/TiO_2_ NWs with an average diameter of 500 nm, oriented vertically on the template. We detail the growth of the NW diameter to 50–170 nm and the uniform NW surfaces of the ZnO/TiO_2_ core/shell arrays with AgCl-doped CdSe QDs in Figure 1c,d, respectively (Figure S4). The ZnO/TiO_2_ NW surfaces could enhance the electrode contact region between the ETL and CH_3_NH_3_PbI_3_ perovskite as well as the adsorption volume between the perovskite and the heterostructure, thereby enhancing the cell performance. The core/shell ZnO/TiO_2_ NWs with AgCl-doped CdSe QDs were analyzed via HR-TEM, as illustrated in Figure 1d, which details a distinct AgCl-doped CdSe QD layer with a diameter of 7–10 nm synthesized on the heterogeneous ZnO/TiO_2_ NWs. The lattice fringes in the HR-TEM image indicated an atomic spacing of 0.259 nm for the center, which was close to the interval spacing between the (002) planes in the hexagonal structure of crystalline ZnO (JCPDS). Furthermore, the distance between adjacent lattice fringes in the shell was 0.234 nm, which corresponded to the interplanar distance of the (004) planes of the anatase structure of TiO_2_. The surface and interfacial electrode regions between the CH_3_NH_3_PbI_3_ perovskite and ZnO/TiO_2_ NWs with AgCl-doped CdSe QDs were significant factors in determining the photoelectric conversion efficiency of photovoltaic devices. It was found that the hetero-structure of the perovskite CH_3_NH_3_PbI_3_ layer enveloping the apex of the ZnO/TiO_2_ NWs had numerous carriers that promoted electron–hole transport for recombination. Additionally, the dense hetero-structure of it was internal to AgCl-doped CdSe QDs of the perovskite layer coating on the surface of ZnO/TiO_2_ NWs.

### 2.2. Photocurrent Density–Voltage (J–V) Characteristics

Figure 2 presents the J–V curves of the perovskite solar cell based on ZnO/TiO_2_ NWs with AgCl-doped CdSe QDs under 100 mW/cm^2^ of AM 1.5 illumination. After the modification of the heterostructured ZnO/TiO_2_ core/shell arrays with AgCl-doped CdSe QDs, the IPCE of the perovskite solar cell based on ZnO/TiO_2_ NWs was enhanced by 3.603%, in comparison with the unmodified device. The improvement in the open-circuit voltage of the perovskite solar cells modified with ZnO/TiO_2_ from 1138 to 1179 mV compared with that of ZnO/TiO_2_ NWs could be attributed to the repression of charge recombination because of the energy level induced by the AgCl-doped CdSe QDs. As illustrated in Figure 2, the indirect adsorption capacity of the CH_3_NH_3_PbI_3_ layers in the opportunity of ZnO/TiO_2_ NWs was less than that at the interface of the ZnO/TiO_2_ NWs owing to the low wettability and adhesion of the CH_3_NH_3_PbI_3_ layer. Furthermore, the abundant of opportunity in ZnO NWs relatively surface in ZnO/TiO_2_ NWs took the recombination region for electron–hole transport, which decreased the performance of the device from 1057 to 1024 mV, as shown in Table 1. Consequently, TiO_2_ covered with AgCl-doped CdSe QDs enhanced the carrier density and uniformity of the CH_3_NH_3_PbI_3_ layer on the apex of the ZnO NWs and improved the adsorption capacity of the perovskite CH_3_NH_3_PbI_3_ layers on the surface of the ZnO NWs. Additionally, the heterogeneous ZnO/TiO_2_ core/shell arrays with AgCl-doped CdSe QDs enhanced the surface contact area between the CH_3_NH_3_PbI_3_ layers and ZnO NWs, thereby increasing the photoelectric conversion efficiency. Furthermore, the absorbance of the perovskite CH_3_NH_3_PbI_3_ layer on the ZnO/TiO_2_ NWs was improved by the introduction of the ZnO/TiO_2_ NW heterostructure, indicating that the photon absorption capacity of the perovskite CH_3_NH_3_PbI_3_ layer on the ZnO/TiO_2_ NWs was better than that of the surface of the ZnO NWs. This was owing to the enormous adsorption volume of the perovskite CH_3_NH_3_PbI_3_ layer on the NWs, which enhanced the photoelectric conversion efficiency and photocurrent density.

### 2.3. IPCE Spectra and Nyquist Plots Studies

**Figure 3:**
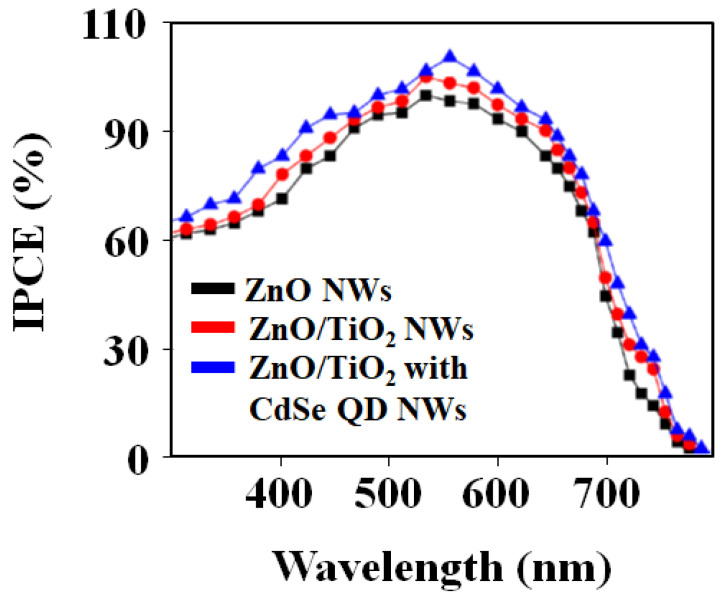
Normalized incident photon conversion efficiency (IPCE) spectra of the PSCs based on ZnO NWs without and with AgCl-doped CdSe quantum dots modification.

Figure 3 presents the IPCE spectra of perovskite solar cells based on ZnO NWs, ZnO/TiO_2_ NWs, and ZnO/TiO_2_ core/shell arrays with AgCl-doped CdSe QDs. The external quantum efficiency of the solar cell with the ZnO/TiO_2_ core/shell arrays with QD modification was higher than that of the unmodified ZnO/TiO_2_ NWs, which was supported by the markedly improved short-circuit current from 21.32 to 22.71 mA/cm^2^ after AgCl-doped CdSe QD modification, as indicated by Table 1 (Figure S5). Furthermore, the AgCl-doped CdSe QD layer could remove the direct point of contact between ZnO/TiO_2_ and the AgCl-doped CdSe QDs absorber optimize. The IPCE spectra of the perovskite solar cells based on ZnO/TiO_2_ NWs with and without the CdSe QD treatment were obtained. The external quantum efficiency of the AgCl-doped CdSe QD perovskite solar cell was higher than that of the cell based on heterogeneous ZnO/TiO_2_ with an increase in the short-circuit current from 21.32 to 22.71 mA/cm^2^ upon QD modification. Moreover, the alteration of the energy level at the interface of the ZnO/TiO_2_ NWs and AgCl-doped CdSe QDs further promoted the transmission and injection of carriers in the ETL and lowered the interfacial electron–hole recombination (Figure S3). Consequently, the overall carrier recombination in the perovskite solar cell was minimized by the heterogeneous AgCl-doped CdSe QDs, resulting in a significant improvement in open-circuit voltage. In addition, to investigate the higher open-circuit voltage and fill factor of the perovskite solar cells based on ZnO/TiO_2_ with AgCl-doped CdSe QDs, EIS measurements were recorded.

**Figure 4:**
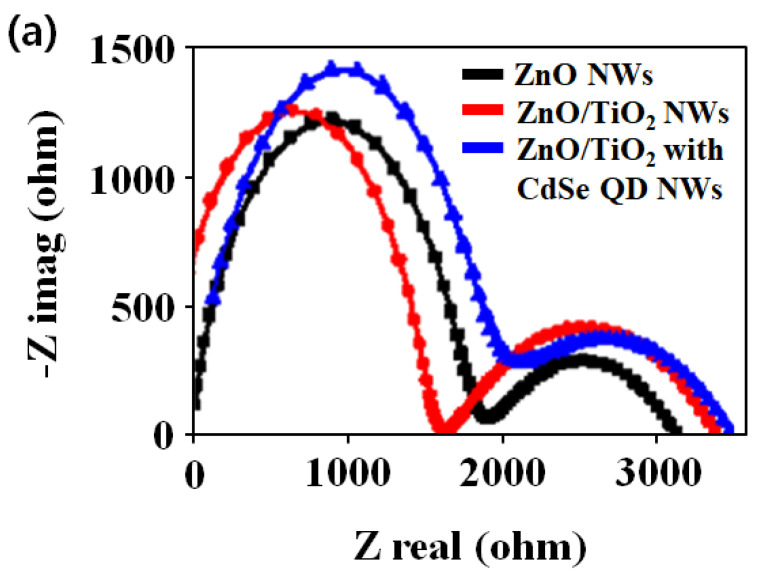

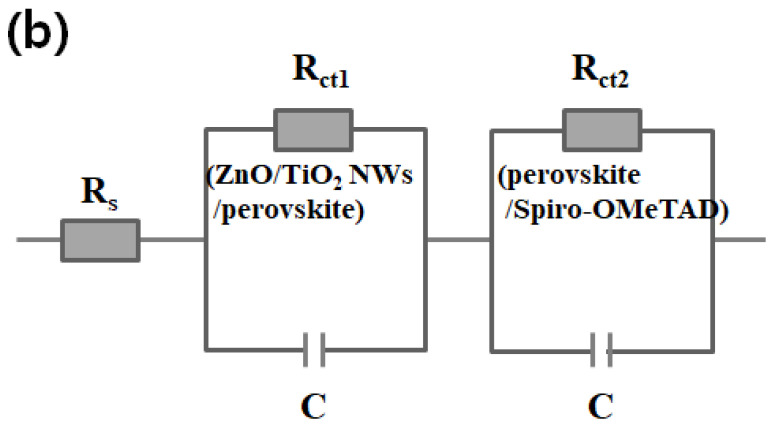
(**a**) Nyquist plots of PSCs based on ZnO/TiO_2_ core/shell arrays with AgCl-doped CdSe quantum dots. (**b**) The equivalent circuit diagram used to fit data.

The spectra in Figure 4a were obtained in the frequency range from 5 to 100 kH_Z_ with an AC amplitude of 5–10 mV under a constant light exposure of 100 mW/cm^2^ to analyze the carrier transport at the surface of the NW/perovskite layers with AgCl-doped CdSe QDs. The equivalent circuit and determined parameters are detailed in Figure 4b (Figure S3). The influence of the core/shell arrays with the AgCl-doped CdSe QD heterostructure on the recombination parameter (*R*_ct1_) at ZnO/TiO_2_ NW arrays with AgCl-doped CdSe QD surface equivalent toward low frequency domain at Nyquist plots is shown [26,27]. *R*_ct1_ of the ZnO/TiO_2_ NW-based perovskite solar cell (625.8 Ω) was less than those of cells based on interfacial ZnO/TiO_2_ core/shell arrays with AgCl-doped CdSe QDs (846.7 Ω), indicating that the QD heterostructure decreased the recombination yield between ZnO/TiO_2_ and CH_3_NH_3_PbI_3_. The modification with AgCl-doped CdSe QDs increased the open-circuit voltage and fill factor, thereby improving *R*_ct1_.

Table 1 summarizes the corresponding photovoltaic parameters. With the ZnO/TiO_2_ NW treatment, the photoelectric conversion efficiency of the perovskite solar cell based on ZnO/TiO_2_ NWs was enhanced by 25.58% compared to that of the device with AgCl-doped CdSe QDs and ZnO/TiO_2_ NWs. The substantially improved open-circuit voltage of the perovskite solar cells treated with ZnO/TiO_2_ NWs from 1024 to 1179 mV compared to that of the AgCl-doped CdSe QDs indicated the repression of recombination response due to the energy obstacle induced by the ZnO/TiO_2_ hetero-structure.

### 2.4. Efficient Photovoltaics Mechanism of Energy Band Diagram

**Scheme 1:**
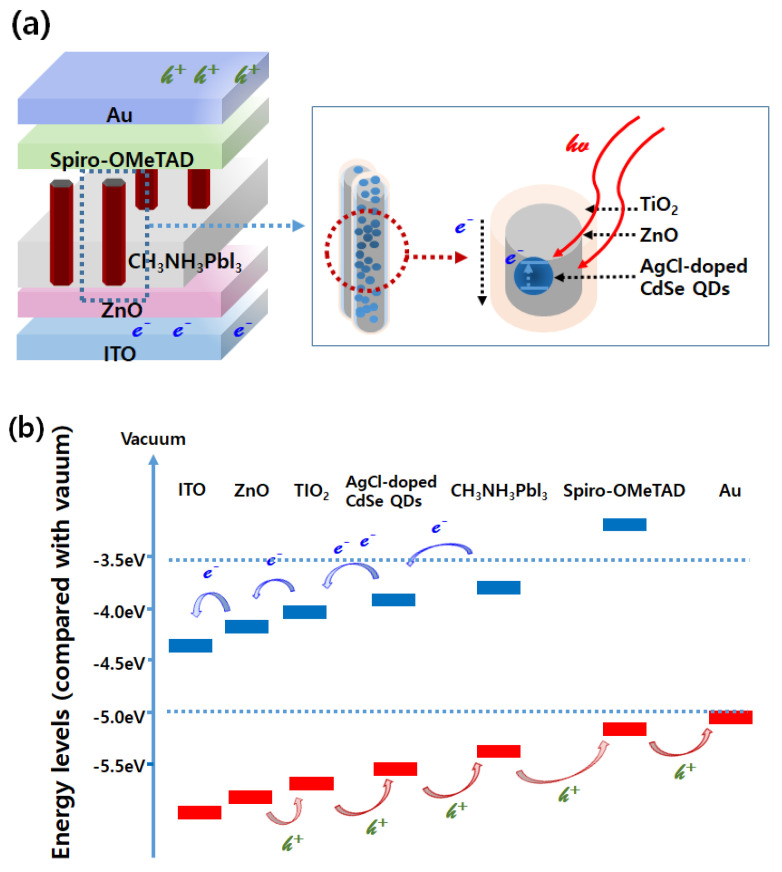
The mechanism for the enhanced photovoltaic activity of the ZnO/TiO_2_ core–shell hybrid arrays with AgCl-doped CdSe quantum dots. (**a**) Device architecture. (**b**) Energy band diagram (work function of different layers).

The core/shell modified ZnO/TiO_2_ NW arrays were fabricated as the ETL in perovskite solar cells (ITO/ZnO/TiO_2_ core/shell arrays with AgCl-doped CdSe QDs | CH_3_NH_3_PbI_3_ | spiro-OMeTAD | Au), as illustrated in Figure 1a (Figure S1). Our fabrication procedure for the perovskite solar cells was based on ZnO/TiO_2_ NWs and the resulting devices were distinctly different from those in previous reports [28,29]. A potential energy diagram of a perovskite solar cell based on ZnO/TiO_2_ NWs with AgCl-doped CdSe QDs is presented in Figure 1b. Carrier transfer preferentially occurs from CH_3_NH_3_PbI_3_ to TiO_2_ with AgCl-doped CdSe QDs and from TiO_2_ to ZnO. To analyze the modification of ZnO/TiO_2_ with AgCl-doped CdSe QDs as an ETL in perovskite solar cells [30,31], an energy band diagram for electron–hole recombination and transport in a solar cell based on ZnO/TiO_2_ NWs with AgCl-doped CdSe QDs is presented in Scheme 1. The heterogeneous perovskite makes direct contact with the ITO substrate because of the interface in the ZnO/TiO_2_ hetero-structure. Owing to the low energy band, the carrier can be supplied into the valence band of the heterogeneous perovskite, resulting in direct surface recombination. Therefore, the electron–hole pair will remain in the ZnO/TiO_2_ heterostructure owing to the inconsistency between the energy levels of ZnO/TiO_2_ and the AgCl-doped CdSe QDs. This will affect the carrier transport in ZnO/TiO_2_ due to the environment of accumulated electron–hole pairs and recombination at the ZnO/TiO_2_ and AgCl-doped CdSe QD absorber surface.

## 3. Materials and Methods

### 3.1. Synthesis of NWs and Fabrication of Device

We report the synthesis of CdSe quantum dots doped with a novel AgCl. We detail the growth of the NW diameter to 50–170 nm and the uniform NW surfaces of the ZnO/TiO_2_ core/shell arrays with AgCl-doped CdSe QDs in Figure 1c,d, respectively (Figures S2 and S4). The ZnO/TiO_2_ NW surfaces can enhance the electrode contact region between the ETL and CH_3_NH_3_PbI_3_ perovskite as well as the adsorption volume between the perovskite and the heterostructure, thereby enhancing the cell performance. The core/shell ZnO/TiO_2_ NWs with AgCl-doped CdSe QDs were analyzed via HR-TEM, as illustrated in Figure 1d that details a distinct AgCl-doped CdSe QD layer with a diameter of 7–10 nm synthesized on the heterogeneous ZnO/TiO_2_ NWs. The lattice fringes in the HR-TEM image indicate an atomic spacing of 0.259 nm for the center. The addition of AgCl causes changes in the morphology of synthesized nanocrystals from spherical nanoparticles to large nanoparticles. In this article, we explain the reasons for the formation of the core/shell of ZnO/TiO_2_ nanowire with AgCl-doped CdSe quantum dots as well as the process. The optical properties of quantum dots are also investigated. Quantum dots (QDs) have attracted considerable attention because of their physical and chemical properties. These properties are dissimilar to those of bulk objects because of the small size of the QDs. QDs can emit at arbitrary emission wavelengths and this property is widely applied in many optical devices. CdSe QDs deserve special attention because of their bright photoluminescence (PL), photostability, and a considerable number of methods for their synthesis. For many of the practical applications of QDs PL is important: for the fabrication of optical fibers, lasers, biolabels, and for the sensibilization of solar cells. The most common way to obtain PL in CdSe QDs is to add some optically active defects that reduce the energy of electron–hole recombination by trapping. Charge carriers could be trapped by both surface defects and volume defects. It is known that doping CdSe QDs with Ag leads to the emergence of a low-energy band [1,2]. However, there are only a few publications devoted to Ag-doped CdSe QDs.

Synthesis of CdSe Nanocrystals: CdSe nanocrystals were prepared by modifying a known procedure. For 3-nm-diameter CdSe nanocrystals, CdO (410.0 mg, 3.2 mmol), hexadecylamine (HDA, 18.54 g, 76.8 mmol), n-dodecylphosphonic acid (DDPA, 1.608 g, 6.4 mmol), and tri-n-octylphosphine oxide (TOPO, 8.096 g, 20.9 mmol) were heated to 90 °C in a 100-mL four-neck round-bottom flask with continuous stirring. The flask was degassed under vacuum (<20 millitorr) and purged with dry N_2_. The degassing process was repeated at least three times to remove water and O_2_. The mixture was then heated to 315 °C under N_2_, and held at that temperature for nearly 30 min until the precursor solution turned clear. After stabilizing the colorless mixture at 280 °C, a mixture of 20 mL of a 0.2 M solution of Se in tri-n-octylphosphine (TOP, 4 mmol) and 0.3 mL of diphenylphosphine (DPP) prepared in a N_2_-filled glove box was rapidly injected into the reaction vessel with continuous stirring, resulting in a temperature drop to ~225 °C. The temperature was then quickly elevated to ~270 °C using a heat gun, and kept at that temperature for ~10 min to facilitate nanocrystal growth. The reaction vessel was cooled to ~90 °C and 40 mL of 1-butanol was added to prevent solidification of the reaction mixture. The nanocrystals were isolated by addition of methanol to induce flocculation, followed by centrifugation. The resulting precipitate yielded nanocrystals with surfaces coated by a mixture of HDA, DDPA, and TOP/TOPO. To remove excess ligands, several additional purification steps were performed, namely the precipitate was redispersed in hexanes and centrifuged. The supernatant, which contained the nanocrystals, was saved, and the precipitate (mostly unreacted hexadecylamine (HDA)) was redispersed in hexanes and centrifuged again to extract more nanocrystals. This process was repeated multiple times (typically 3) until all the possible nanocrystals were extracted from the precipitate into the supernatant. The dispersion was then stored overnight in a freezer (−20 °C). During this time, excess surfactant precipitated out of the dispersion and was removed by centrifugation. The supernatant was then filtered through a 0.2 µm polytetrafluoroethylene (PTFE) syringe filter, reagent alcohol was added, and the solution was centrifuged. Multiple iterations (in general two more cycles) of redispersion and precipitation using hexanes and reagent alcohol were done to obtain pure CdSe nanocrystals. Finally, the nanocrystals, isolated as solid centrifuge pellets, were dried under vacuum, dispersed in toluene, filtered through a 0.2 µm PTFE syringe filter to obtain a stable colloidal dispersion, and stored under ambient conditions until needed.

Doping of CdSe Nanocrystals with Ag: To incorporate Ag, a typical exchange reaction heated 10 mL of a 5 mg/mL dispersion of CdSe nanocrystals in toluene to ~65 °C in a glass vial with continuous stirring. An amount of 1 mL of 0.1 M ethanolic AgNO_3_ was combined with 1.5 mL of TOP and then added to the rapidly stirring dispersion. After ~5 min the reaction was quenched with ~10 mL of ethanol. The precipitated nanocrystals were isolated by centrifugation and then dispersed and isolated several times with hexanes and ethanol, respectively, to obtain a clean product. This process generates nanocrystals with ~1 Ag per particle on average. We used a modification of the oleate method to prepare Ag-doped CdSe QDs using AgCl as a precursor of Ag. We focused our attention on the two origins of trap states: halides and silver. The cation exchange reaction in nanocrystals, investigated mainly with Ag^+^ ion in this study, can easily be extended to exchange with other cations. For example, CdSe nanocrystals can be successfully transformed into CuSe and PbSe nanocrystals through the cation exchange reaction with Cu2^+^ and Pb^2+^ ions, respectively, under ambient conditions.

ZnO NWs were grown by a investigated SiO_2_ soft mask process. Firstly, ITO substrates (20 mm × 20 mm × 3 mm) were washed by acetone and de-ionized water, and then dried with nitrogen. The zinc acetate dihydrate with accurate quantity was dissolved in ethanol. Secondly, the precursor was spin coated onto the ITO, and the substrates were then annealed. This procedure was iterated five times to augment the layer, and the substrates were annealed in atmosphere at 380 °C for 20 min. Lastly, zinc oxide layers were derived, which were conjugated as the SiO_2_ template for the growth of ZnO NWs. Then, the PVP–Zn^2+^ composite solution treatment was arranged by zinc acetate dihydrate with polyvinylpyrrolidone solution under magnetic stirring at 65 °C. The ammonia (NH_3_·H_2_O, 0.95 g/mL) was induced to optimize the pH number of the PVP–Zn^2+^ composite liquid solution to a determine figure. The zinc oxide structure were laid side on the hybrid system in the vessels which comprised the composite liquid solution as the growth facility. Consequentially they were put in a tube furnace in an oxygen atmosphere. The temperature of tube furnace was raised from 25 °C to 450 °C with a heating rate of 10 °C/min and maintained at 450 °C for 60 min during the anneal procedure before changing to 300 K. The deposition was conducted to supply the AgCl-doped CdSe quantum dots on ZnO NWs. TiO_2_ crystals without the atomic defect 1st shell structure were covered on the interface of ZnO NWs by a sol–gel process. Then, the ITO substrates coated with ZnO NWs were switched in the system, which contained titanium but oxide structure with isopropanol as liquid solution in accurate capacity ratio with magnetic stirring. DI-water and titanium precursor liquid solvent were incorporated in a certain H2O/Ti(OC4H9)4M ratio to facilitate the hydrolysis change and condensation parameter to present TiO_2_ particles on the nanowire interface. The deposition was conducted to supply the methyl-ammonium lead halides into ZnO/TiO_2_ NWs. Firstly, lead iodide was redissolved in *N*,*N*-dimethylformamide at accurate density under stirring at 75 °C. The liquid solvent was maintained at 75 °C for the process. Then the lead iodide liquid solvent interpenetrated into the ZnO/TiO_2_ NWs by the spin-coating method, and the cells were dried at 75 °C for 60 min. After cooling down to the 300 K, the cells were bathed into a solution of CH_3_NH_3_I in isopropyl alcohol (20 mg/mL) for a half h, and then the CH_3_NH_3_I solution was spin-coated onto the cells. Then, the structure was dried at 75 °C for a half h. The hole transport layer (HTL) was investigated by spin coating 80 µL co-doped spiro-MeOTAD liquid solvent on the methylammonium lead halides layer. Finally, a 100 nm thickness gold film was thermal evaporated on the hole transport layers to structuralize the rear electrode.

### 3.2. Characterization

Morphological and nano-structural analyses were performed using scanning electron microscopy (SEM) (Jeol JSM-6210) and high-resolution transmission microscopy (HRTEM) (FEI Tecnai F30 S-Twin), respectively. During cross sectional TEM imaging, samples were milled with 10- to 30-kV gallium ions accelerated by using a focused ion beam (FIB, FEI NOVA 200) in the dual-beam mode. The photocurrent density–voltage (J–V) curves of the devices were obtained by an electrochemistry workstation (CHI660D, 0.1 MHz to 100 Hz) under irradiation of simulated solar light (AM 1.5 G, 100 mW/cm^2^). The photon density of the illumination source was calibrated by using a power meter. Electrochemical impedance spectra were measured by the CHI-660E electrochemical workstation using the AC impedance method of light illumination of 100 mw/cm^2^. The applied initial voltage was set at the open-circuit voltage (Voc) of the PSC.

## 4. Conclusions

In summary, we designed CdSe QDs with AgCl dopants that were fabricated by an oleate colloidal process. The performance of the perovskite solar cells was determined on the basis of ZnO/TiO_2_ NWs. The band diagram contains a gradational component due to the arrangement of an *n*-type perovskite structure. The recombination parameter (*R*_ct1_) was improved by titanium shell supplementation with AgCl-doped CdSe QDs, which improved the open-circuit voltage, fill factor, and adsorption volume of CH_3_NH_3_PbI_3_ on the NWs. The enhanced cell durability was also attributable to TiO_2_ supplementation. A maximum photoelectric conversion efficiency of 15.12% was attained in an atmospheric environment, which the comparatively humid circumstance in the manufacture procedure. The mechanisms of electron–hole recombination and transport in the perovskite solar cells based on the ZnO/TiO_2_ core/shell arrays evidenced the advantages of the ZnO/TiO_2_ core/shell with AgCl-doped CdSe QD arrays as an effective ETL in photovoltaic devices. This study demonstrated a simple approach to restraining non-radiative recombination in core/shell arrays to further maximize the performance of perovskite solar cells toward highly efficient photovoltaics.

## Figures and Tables

**Table 1:** Photovoltaic parameters of PSCs based on ZnO/TiO_2_ core/shell arrays with AgCl-doped CdSe quantum dots under 100 mW/cm^2^ of AM 1.5 illuminations.

Samples	Voc (V)	Jsc (mA cm^−2^)	FF (%)	H (%)
ZnO NWs arrays	1.024	21.32	0.631	12.04
ZnO/TiO_2_ core/shell arrays	1.057	22.01	0.674	13.25
ZnO/TiO_2_ core/shell arrays with QDs	1.138	22.18	0.697	14.34
ZnO/TiO_2_ core/shell arrays with AgCl doped QDs	1.179	22.71	0.712	15.12

## Supplementary Materials

The following are available online. Figure S1: Top-view SEM images of perovskite thin films based on different ratios of Li doping; Figure S2: Steady-state PL spectra of perovskite with core/shell of ZnO/TiO_2_ nanowire arrays; Figure S3: EIS parameters of perovskite solar cells; Figure S4. HR-TEM images of AgCl-doped CdSe quantum dots; Figure S5: Photovoltaic parameters of PSCs based on ZnO/TiO_2_ core/shell arrays with AgCl-doped CdSe quantum dots under 100 mW/cm^2^ of AM 1.5 illuminations.
