# Supplementary material for: High-Performance Core/Shell of ZnO/TiO2 Nanowire with AgCl-Doped CdSe Quantum Dots Arrays as Electron Transport Layer for Perovskite Solar Cells
Source: Molecules. 2020 Aug 31;25(17):3969. doi: 10.3390/molecules25173969 (PMC7504724; doi:10.3390/molecules25173969)
Supplement: Supplementary file 1 [file molecules-25-03969-s001.pdf]

Supplementary Information

High-performance core/shell of ZnO/ TiO_2 nanowire with AgCl-doped CdSe quantum dots arrays as electron transport layer for perovskite solar cells

Figure S1. Top-view SEM images of perovskite thin films based different ratios of Li doping.

Top-view SEM images of the perovskite layer based on ZnO under different atmosphere, the scale bar indicates 1 μm.

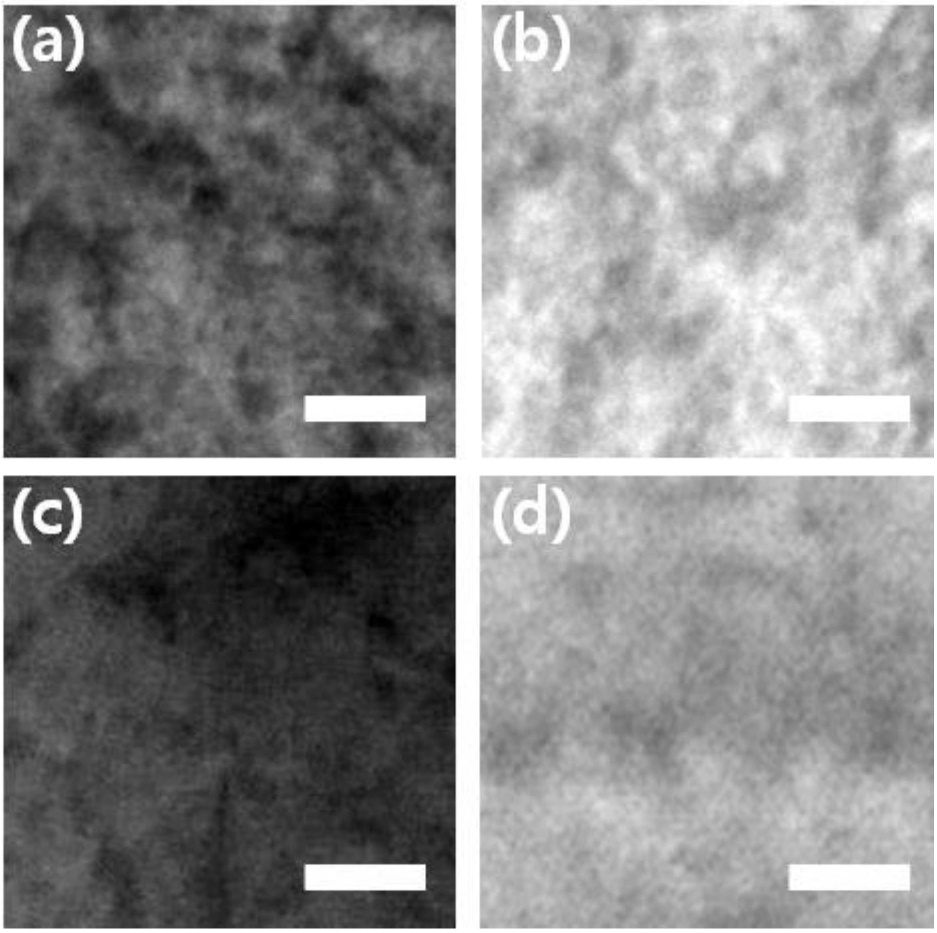

Figure S2. Steady-state PL spectra of perovskite with core/shell of ZnO/ TiO_2 nanowire arrays.

PL spectra of perovskite with core/shell of ZnO/ TiO_2 nanowire with AgCl-doped CdSe quantum dots arrays.

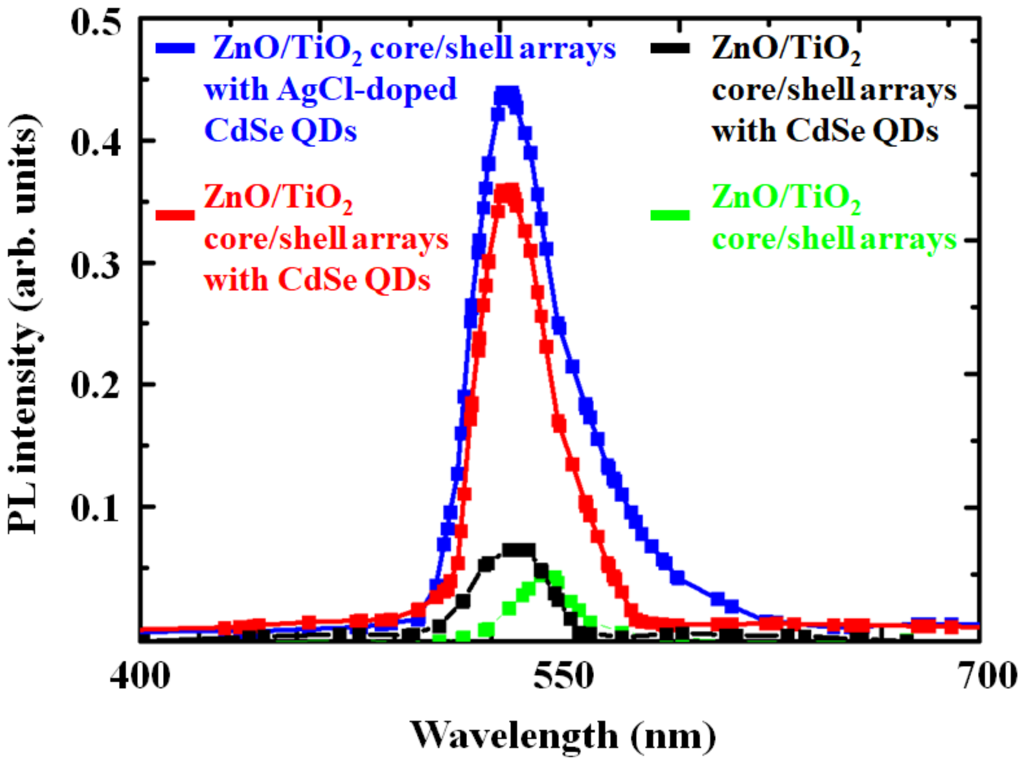

Figure S3. EIS parameters of perovskite solar cells.

Nyquist plots of PSCs based on ZnO/TiO2 core/shell arrays with AgCl-doped CdSe quantum dots.

Devices	R_s (ohm)	R_{ct1} (kohm)	R_{ct2} (kohm)
ZnO/TiO_2 core/shell arrays	74.0	125.2	24.6
ZnO/TiO_2 core/shell arrays with AgCl-doped CdSe quantum dots	32.3	143.3	46.2

Figure S4. HR-TEM images of AgCl-doped CdSe quantum dots.

The HR-TEM images of AgCl-doped CdSe quantum dots, the scale bar indicates 10 nm.

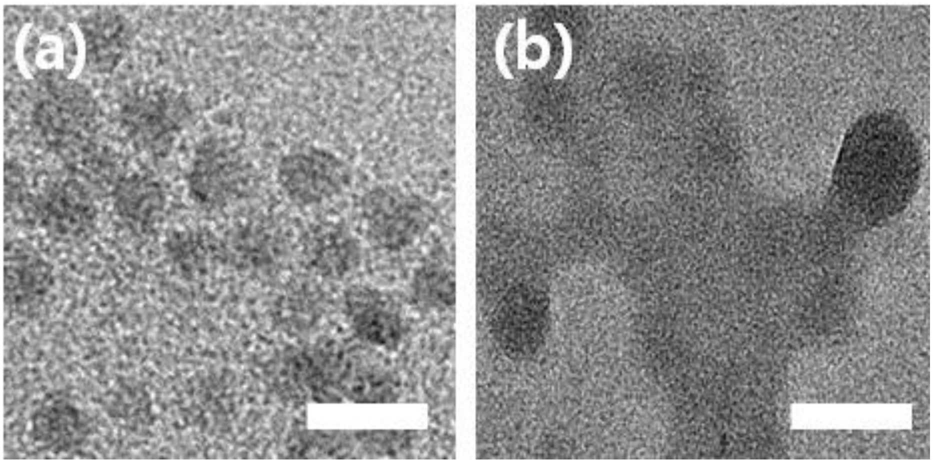

Figure S5. Photovoltaic parameters of PSCs based on ZnO/TiO2 core/shell arrays with AgCl-doped CdSe quantum dots under 100 mW/cm2 of AM 1.5 illuminations.

Evolution of the J-V parameters of the Zn:SnO2 based devices stored in the ambient air with a relative humidity of ~20%.

Time (h)	V_{oc} (V)	J_{sc} (mA cm^{-2})	FF (%)	η (%)
**0**	1.132	21.94	0.679	14.96
**24**	1.146	22.35	0.689	15.02
**48**	1.178	22.16	0.713	15.09
**72**	1.164	22.34	0.698	15.11

References

[1] Ferekides CS, Mamazza R, Balasubramanian U, Morel DL. Transparent conductors and buffer layers for CdTe solar cells. Thin Solid Films 2005;480:224–9.

[2] Islam MA, Rahman KS, Sobayel K, Enam T, Ali AM, Zaman M, et al. Fabrication of high efficiency sputtered CdS: O/CdTe thin film solar cells from window/absorber

layer growth optimization in magnetron sputtering. Sol Energy Mater Sol Cells 2017;172:384–93.

[3] Takamoto T, Agui T, Kurita H, Ohmori M. Improved junction formation procedure for low temperature deposited CdS/CdTe solar cells. Sol Energy Mater Sol Cells 1997;49(1–4):219–25.

[4] Minami T, Nishi Y, Miyata T. Effect of the thin Ga2O3 layer in n+-ZnO/n-Ga2O3/p-Cu2O heterojunction solar cells. Thin Solid Films 2013;549:65–9.

[5] Chiang HQ, Wager JF, Hoffman RL, Jeong J, Keszler DA. High mobility transparent thin-film transistors with amorphous zinc tin oxide channel layer. Appl Phys Lett 2005;86(1):013503.

[6] Patil MA, Mujawar SH, Ganbavle VV, Rajpure KY, Deshmukh HP. Synthesis and characterization of zinc stannate thin films prepared by spray pyrolysis technique. J Mater Sci: Mater Electron 2016;27(12):12323–8.

[7] Rajachidambaram JS, Sanghavi S, Nachimuthu P, Shutthanandan V, Varga T, Flynn B, et al. Characterization of amorphous zinc tin oxide semiconductors. J Mater Res 2012;27(17):2309–17.

[8] Kim DH, Cho NG, Kim HG, Choi WY. Structural and electrical properties of indium doped ZnO thin films fabricated by RF magnetron sputtering. J Electrochem Soc 2007;154(11):H939–43.

[9] Caruge JM, Halpert JE, Wood V, Bulović V, Bawendi MG. Colloidal quantum-dot light-emitting diodes with metal-oxide charge transport layers. Nat Photon 2008;2(4):247.

[10] Islam MA, Hossain MS, Aliyu MM, Karim MR, Razykov T, Sopian K, et al. Effect of CdCl2 treatment on structural and electronic property of CdTe thin films deposited by magnetron sputtering. Thin Solid Films 2013;546:367–74.

[11] Satoh K, Kakehi Y, Okamoto A, Murakami S, Uratani F, Yotsuya T. Influence of

oxygen flow ratio on properties of Zn2SnO4 thin films deposited by RF magnetron sputtering. Jpn J Appl Phys 2004;44(1L):L34.
[12] Islam MA, Khandaker MU, Amin N. Effect of deposition power in fabrication of highly efficient CdS: O/CdTe thin film solar cell by the magnetron sputtering technique. Mater Sci Semicond Process 2015;40:90–8.
[13] Zhang SB, Wei SH, Zunger A. Intrinsic n-type versus p-type doping asymmetry and the defect physics of ZnO. Phys Rev B 2001;63(7):075205.
[14] Minami T, Nanto H, Takata S. Highly conductive and transparent zinc oxide films prepared by rf magnetron sputtering under an applied external magnetic field. Appl Phys Lett 1982;41(10):958–60.
